# Combining structured exercise with a low-energy diet to attenuate lean mass loss in South Asian adults living with type 2 diabetes: the COMBINE randomised trial protocol

**DOI:** 10.1136/bmjopen-2025-110459

**Published:** 2026-03-23

**Authors:** Franciskos Arsenyadis, Joseph Henson, Matthew McCarthy, Dimitris Papamargaritis, James A King, Emma Redman, Gráinne Whelehan, Thomas Wilkinson, Jack Sargeant, Alex Rowlands, Normand Boulé, Kaberi Dasgupta, Gerry P McCann, Melanie J Davies, Kamlesh Khunti, Pratik Choudhary, Thomas Yates

**Affiliations:** 1 University of Leicester Diabetes Research Centre, Leicester, UK; 2 NIHR Leicester Biomedical Research Centre, Leicester, UK; 3 Diabetes Research Centre, NIHR Leicester Biomedical Research Centre, Leicester, UK; 4 National Centre of Sport and Exercise Medicine, Loughborough University, Loughborough, UK; 5 Leicester Diabetes Centre, University Hospitals of Leicester NHS Trust, Leicester, UK; 6 Faculty of Kinesiology, Sport, and Recreation, University of Alberta, Edmonton, Alberta, Canada; 7 Divisions of Clinical Epidemiology and Internal Medicine, McGill University Health Centre, Montreal, Québec, Canada; 8 University of Leicester Department of Cardiovascular Sciences, Leicester, UK; 9 NIHR Applied Research Collaboration East Midlands, Leicester, UK

**Keywords:** Diabetes Mellitus, Type 2, Obesity, NUTRITION & DIETETICS, Exercise

## Abstract

**Introduction:**

The global prevalence of type 2 diabetes (T2D) is rising and disproportionately affects South Asian adults, including those in the United Kingdom. South Asians develop T2D at a higher rate and at a younger age than their white British counterparts, at a lower body mass index. Active efforts to reduce adiposity can improve glycaemic control and in some cases achieve T2D remission. However, a substantial proportion of lean mass is lost while achieving weight loss, which may have physiological and metabolic consequences, affecting long-term health outcomes and quality of life for people living with T2D and obesity. We are examining the impact of a combined low energy diet and supervised exercise intervention versus a low energy diet alone for the preservation of lean mass in an understudied South Asian population living with T2D and excess adiposity.

**Methods and analysis:**

This prospective, randomised, two-arm parallel-group, open-label, blinded-endpoint trial is being conducted in Leicester, UK. 36 South Asian adults aged 40–65 years within 10 years of T2D diagnosis and not on insulin therapy will be enrolled. Both intervention arms will receive an 800–900 kcal/day low energy diet for 12 weeks. Those randomised to the exercise group will additionally receive a mixture of supervised and home-based resistance and aerobic exercise training three times per week. The primary outcome is the difference in the change of lean mass between groups measured using dual-energy X-ray absorptiometry at baseline and 12 weeks and will be analysed using linear regression modelling.

**Ethics and dissemination:**

The trial was approved by the NHS research ethics service (23/WM/0201). All participants will provide informed consent prior to enrolment, and the study will be conducted in accordance with the Declaration of Helsinki. Findings will be shared widely (publications, presentations, press releases, social media platforms) and will inform an effectiveness trial.

**Trial registration number:**

ISRCTN11175684.

**STRENGTHS AND LIMITATIONS OF THIS STUDY**

- Participants are randomised to two intervention arms with comparable prescribed energy restriction, differing only by the inclusion of structured exercise.
- Lean mass and body composition are assessed using dual-energy X-ray absorptiometry, with complementary physiological and imaging measures collected using predefined procedures.
- Physical function, fitness and strength are measured using objective, validated assessments, including cardiopulmonary exercise testing, dynamometry and performance-based functional tests.
- The open-label design may allow for unmeasured changes in habitual behaviours outside the prescribed interventions, despite blinded endpoint assessment.
- The single-centre design, short intervention period and limited sample size may limit generalisability and lead to type 2 errors in secondary outcomes.

## Background

 Increasing numbers of adults are developing type 2 diabetes (T2D), mirroring the global rise in excess adiposity (overweight and obesity).^1^ Living with T2D increases the risk of developing associated comorbidities, with micro and macrovascular complications a common and debilitating presentation affecting physical and mental health and increasing the risk of premature mortality.^2 3^ The South Asian subcontinent is projected to account for some of the highest rates of new cases by 2045 (124.9, 62.2 and 22.3 million in India, Pakistan and Bangladesh, respectively).^1^ These high rates of T2D are reflected in the South Asian diaspora living in the United Kingdom, who are at a higher risk of T2D at a markedly lower body mass index (BMI) and younger age than white Europeans.^4^,^7^ This heightened susceptibility underscores the importance of identifying effective lifestyle intervention strategies for South Asian populations.

Remission of T2D through lifestyle intervention, defined as return of glycated haemoglobin (HbA1c) to a below-diagnostic threshold without glucose-lowering therapies,^8^ is a research area highlighted as a patient priority with mounting scientific interest.^9^ Remission through diet has been demonstrated in the landmark DiRECT trial,^10^ which showed that a remission rate of over 40% at 1 year was achievable in recently diagnosed T2D through significant and sustained weight loss. This weight loss (approximately 10% of starting bodyweight) was achieved through a low energy diet (LED), offering approximately 850 kcal/day with 0.55 g/kg/day of protein through meal replacement products (MRP).

While this significant weight loss can markedly improve metabolic outcomes and physical function, it also has implications for lean mass (LM) losses, which are typically seen with 20% to 30% of weight loss.^11^ Skeletal muscle, the major component of LM, is critical for mobility, independent activities of daily life and a major storage site for glucose. Protecting from LM loss in high-risk populations undertaking intentional weight loss may be desirable in order to optimise physical function and reduce the risk of frailty, fragility fractures and impaired metabolic health.^12 13^ This is particularly important for individuals with an unfavourable fat-to-LM ratio. South Asians, for example, carry on average 3–4 kg less LM than white Europeans at a given fat mass and have a higher ratio of fat to LM^14^,^17^ and liver fat when matched for BMI.^18^ The lower LM is largely due to lower appendicular LM (a proxy for skeletal muscle mass),^14 15^ which is crucial for mobility and independence in daily life. Protecting even 1 kg of LM could aid physical function and strength, counteracting some of the age-related declines, estimated to be 1.5 kg per decade^19 20^ in weight-stable middle-aged individuals, and the accelerated physiological decline observed in T2D.^20 21^

Resistance exercise holds promise for preserving muscle while enhancing relative strength and physical function—benefits that could support those typically seen during intentional weight loss.^22^ Additionally, it may help to counteract the long-term negative effects of ageing and diabetes on bone health,^23^ while also mitigating the adverse effects of energy restriction on bone health by applying repeated and progressive biomechanical strain, which stimulates bone remodelling and offsets the reduced loading with weight loss.^24^

Complementing resistance training, aerobic training offers distinct metabolic advantages. Beyond its well-established role in improving cardiorespiratory fitness, aerobic training can also improve muscle quality by stimulating mitochondrial biogenesis^25^ and reducing intermuscular fat^26^ and insulin resistance.^27^ However, despite these benefits, aerobic exercise alone does not stimulate muscle growth or preserve LM during caloric restriction to the same extent as resistance exercise.^28^ This indicates that a combined approach, integrating both resistance and aerobic training, may support the benefits of energy restriction while minimising the unintended side effects of intentional efforts to reduce adiposity.

The LITOE study,^22^ a 26-week head-to-head trial comparing aerobic versus resistance exercise or both during 10% matched (diet-induced) weight loss in older (≥65 years) adults with obesity, showed that resistance exercise, alone or in combination with aerobic exercise, resulted in the greatest benefits to physical function and preservation of LM. Furthermore, the combined exercise group also showed the most significant improvements in insulin sensitivity and reductions in visceral adipose tissue.

Despite these findings, the effects of supervised exercise performed alongside an MRP-based LED, which induces a greater and more controlled energy deficit than standard reduced-calorie diet interventions, while being actively investigated in early onset T2D,^29^ have yet to be examined in South Asian adults. This is particularly relevant for South Asian individuals with T2D, who may benefit from the dual therapeutic effects of LM preservation and metabolic improvement (ie, increased insulin sensitivity and reduced visceral adipose tissue) during attempts at T2D remission.

While current guidelines recommend a combined approach of resistance and aerobic exercise to support musculoskeletal and metabolic health, this strategy has not been specifically tested in the context of a structured LED weight loss intervention for South Asians. This is particularly important given the increased susceptibility of South Asians to T2D-related long-term conditions, which is further compounded by their under-representation in clinical trials.^30 31^

The COMBINE trial addresses this gap by recruiting middle-aged South Asian adults living in the UK who wish to attempt T2D remission, while aiming to minimise LM losses through an LED combined with supervised resistance and aerobic exercise training.

### Aims

The aim of the COMBINE trial is to investigate whether a structured exercise training programme combined with an MRP-based LED minimises LM loss more effectively than an MRP-based LED alone over a 12-week period in middle-aged South Asian adults (age ≥40 and ≤65 years) with T2D. LM changes will be evaluated using dual-energy X-ray absorptiometry (DXA). It is hypothesised that the combined intervention, comprising structured exercise training and an MRP-based LED, will significantly mitigate the anticipated reductions in LM.

Secondary objectives (outlined in table 1) will examine the effects of the intervention on a range of physiological responses associated with the primary outcome. These include cardiorespiratory fitness, physical function and strength, anthropometry and body composition, skeletal muscle ultrasound imaging for assessment of size (muscle cross-sectional area, thickness, volume, subcutaneous fat), texture (echo intensity) and architecture (fibre pennation angle), and resting metabolic rate through precise quantification of oxygen consumption and carbon dioxide production. Additionally, the study will examine key cardiometabolic outcomes related to T2D, including mean glucose levels, glycaemic variability, time spent within, time spent above and below target glucose ranges using continuous glucose monitoring, diabetes remission and associated cardiometabolic risk factors using pathology measures. Lifestyle behaviours influenced by the intervention will also be investigated, including physical activity levels, sedentary behaviour, sleep, dietary intake and patient-reported outcomes (including anxiety, depression and quality of life).

**Table 1:** Outcomes

	Baseline	Week 12
**Primary outcome**		
Lean body mass (DXA)	X	X
**Key secondary outcomes**		
DXA-derived body composition measures		
Appendicular lean mass (arms, legs and total)	X	X
Total and percentage fat mass	X	X
Visceral adipose tissue	X	X
Subcutaneous adipose tissue	X	X
Total bone mineral density and bone mineral content	X	X
Regional bone mineral density (hip, femoral neck, lumbar spine, trabecular bone)	X	X
Other anthropometric assessments		
Weight and body mass index	X	X
Waist and neck circumference	X	X
Muscle ultrasound		
Quadriceps muscle diameter, volume and quality	X	X
Main fitness measure		
VO_2_ peak (absolute, relative to lean body mass, relative to overall body mass)	X	X
Other exercise stress test measures		
VCO_2_ peak, maximum gradient achieved	X	X
Physical function and strength		
Handgrip strength	X	X
Isometric and isokinetic quadriceps strength (Biodex)	X	X
Short Physical Performance Battery	X	X
Sit-to-stand 60	X	X
**Other secondary outcomes**		
Cardiometabolic and pathology measures		
Diabetes remission at 12 weeks		X
Haemoglobin A1c	X	X
Hypertension remission, systolic and diastolic blood pressure, heart rate	X	X
Renal function measures		
Creatinine and estimated glomerular filtration rate	X	X
Urine albumin to creatinine ratio	X	X
Hepatic function measures		
Alkaline phosphatase, alanine transaminase, aspartate transaminase, gamma-glutamyl-transferase	X	X
Total cholesterol, HDL, LDL, triglycerides	X	X
Indirect calorimetry		
Resting metabolic rate	X	X
Continuous glucose monitoring		
Mean glucose levels, variability and time within, above and below range	X	X
Optional mixed-meal tolerance test		
Fasting and post-prandial insulin and glucose levels	X	X
Overall health state		
EuroQuol group 5-Dimensional 5-Level Questionnaire	X	X
WHO Disability Assessment Schedule 2.0	X	X
Dyspnoea scale	X	X
Depression, anxiety and distress		
Hospital Anxiety and Depression Scale and Diabetes Distress Scale	X	X
Dietary variables		
Total energy and macronutrient intake (protein, carbohydrates, lipids)	X	X
Selected carbohydrate (total sugars, fibre) and lipid (saturated, monounsaturated, polyunsaturated) types, relevant micronutrients and alcohol	X	X
Accelerometer-based physical activity measures and sleep (daily average)		
Steps, overall acceleration and intensity gradient metric	X	X
Minutes for each of sedentary, light and moderate to vigorous physical activity	X	X
Sleep time, duration of night, sleep efficiency (sleep time/duration of night)	X	X
Intervention acceptability		
Completion rates and percentage weight loss achieved		X

DXA, dual-energy X-ray absorptiometry; HDL, high-density lipoprotein; LDL, low-density lipoprotein.

## Methods

### Design

Prospective, randomised, parallel-arm, open-label, blinded-endpoint efficacy trial.

### Setting

Leicester, UK.

### Trial time frame

The first participant was recruited on 14 March 2024. Recruitment, intervention and data analysis are aimed to be completed by 30 December 2025.

### Eligibility criteria

South Asian adults aged 40–65 years, with a clinical diagnosis of T2D and a BMI of ≥25 kg/m^2^, diagnosed between 3 months and 10 years, with HbA1c between 6.5% and 10.0% (if not on glucose-lowering medication) or 6.0%–10.0% (if on such medications), and not currently using insulin. In this study, ethnicity is assessed through participant self-declaration, consistent with standard data capture practice in UK health research and clinical datasets. Age-based inclusion criteria were established to promote a homogenous adult T2D group in relation to the primary outcome given age is an important determinant of LM, with geriatric or young T2D populations likely to present with differences in metabolic and physiological phenotypes, and to capture the majority of newly diagnosed T2D^32^ maximising potential for T2D remission. The use of intensive weight loss interventions at lower BMI thresholds remains an area of ongoing debate. For this efficacy-focused physiological trial, a minimum BMI threshold of ≥25 kg/m^2^ was selected to ensure participants had sufficient excess adiposity to safely undergo an LED and to minimise heterogeneity in diabetes phenotype, body composition and metabolic risk. Eligible individuals will be randomly assigned to one of two arms (described below). Full eligibility criteria are provided in table 2. Before any screening procedures are conducted, participants will provide written informed consent (online supplemental file 1) during a visit with a healthcare professional.

**Table 2:** Eligibility criteria

Inclusion criteria	
Ethnicity	South Asian ethnicity (self-declared ethnicity of self).
Age	Aged ≥40 and ≤65 years.
Diabetes status	Clinically coded diagnosis of T2D between 3 months and 10 years previously.
Glycaemic control	HbA1c 6.5% (48 mmol/mol) to 10% (86 mmol/mol) if not taking glucose-lowering medication; 6% (42 mmol/mol) to 10% (86 mmol/mol) if taking glucose-lowering medication.
Diabetes medication(s)	Treatment stable; no significant change to glucose-lowering regimen in the preceding 3 months, as determined by a study investigator.
Body mass index	≥25 and ≤45 kg/m^2^.
Weight trajectory	Self-reported stable weight over the previous 6 months (<± 5% of bodyweight).
Consent	Able to provide informed consent. Able to understand written and spoken English or willing to use the University Hospitals of Leicester professional interpreter service. Able to take part in structured exercise training requiring the lower limbs (eg, able to walk without assists or impairment).
Willingness	Willingness and availability to participate in the proposed interventions to which they may be assigned, including attendance of intervention visits such as exercise sessions and adoption of LED which requires abstinence from alcohol. Willingness to self-monitor glucose, blood pressure and weight.
**Exclusion criteria**	
Diabetes status	Individuals with type 1, gestational or monogenic diabetes mellitus.
Insulin use	On insulin therapy (note: No COMBINE participant will be on insulin at baseline as this is an exclusion criterion. An exception may be made for women who are on insulin because of its lack of teratogenic effects rather than because of inability to control glycaemia on oral agents alone. On oral or injected steroids. On weight loss medications (not including glucose lowering medication).
Kidney function	eGFR<45 mL.min^−1^ per 1.73 m^2^.
Surgical history	Previous bariatric surgery.
Eating disorder	Self-reported or diagnosed eating disorder.
Diet	Self-reported milk protein allergy or other allergy or dietary practice that prohibits the use of meal replacement products.
Cardiovascular health	Previous myocardial infarction, stroke, amputation secondary to T2D/peripheral vascular disease, or admission due to CVD-related event within 12 months. Previous clinically diagnosed atrial fibrillation. Previous clinically diagnosed heart failure. Pacemaker or implantable cardioverter defibrillator (ICD). Presenting with cardiac abnormalities during the exercise ECG test (inclusive of very high blood pressure).
Eye health	Currently receiving or requiring active treatment for retinopathy.
MMTT	Severe Intolerance or unwillingness/inability to undertake Mixed Meal Tolerance Test (severity of intolerance to be assessed by a member of the research team during screening visit).
Other	Current participation in another research study with investigational medical product. Currently participating in a weight reduction programme in addition to routine care. Conditions that could impact weight (ie, active malignancy/treatment in past year, pregnancy, lactation, planning to become pregnant in the next 8 months). Drugs or conditions thought by the investigators to have significant impact on the study protocol or outcomes. Substance abuse. The requirement for alcohol abstinence during the initial 12 weeks will make it unlikely that individuals with alcohol dependence will enrol. Substance abuse will be queried.

CVD, cardiovascular disease; eGFR, estimated glomerular filtration rate; HbA1c, glycated haemoglobin; LED, low energy diet; MMTT, mixed meal tolerance test; T2D, type 2 diabetes.

### Recruitment

Recruitment will be primarily through diabetes clinics and primary care. To enhance outreach and engagement, supplementary recruitment methods will also include targeted publicity via social media platforms.

### Measurements

All baseline assessments will be performed prior to randomisation, with follow-up assessments scheduled at 12 weeks±4 weeks following intervention initiation. The primary and secondary outcomes deriving from these measurements are outlined in table 1.

#### Body composition and anthropometric measures

Weight will be measured to the nearest 0.1 kg and height, waist and neck circumference to the nearest 0.1 cm. DXA (GE Lunar) will serve as the primary method for assessing body composition measures, including total fat mass (including visceral and subcutaneous fat), LM (including appendicular LM) and total and regional bone mineral density.

#### Muscle ultrasonography

Muscle ultrasound of the rectus femoris (RF) and vastus lateralis (VL) will be performed using a portable ultrasound system. With the participants’ supine and their knee fully extended, landmarks of the upper and lower RF are identified and the distance between the two sites will be measured. Parameters of interest (assessed at 50% of femur length) include muscle cross-sectional area, muscle and subcutaneous fat thickness, fibre angle pennation and texture analysis (acquired postscan using frame capture software ‘Image j’). Muscle and subcutaneous thickness of the VL are assessed laterally at the RF measurement site.

#### Physical fitness, function and strength measures

Following a 3 min warm-up at a fixed speed of 3 km/hour and 0% gradient, participants will undergo a graded treadmill exercise test. The protocol involves a constant walking speed with incremental increases in gradient (1% each minute). Respiratory gas exchange will be monitored to measure oxygen consumption and carbon dioxide production. The test continues until one of the following termination criteria is met; 100% of age-predicted maximum heart rate (85% if using beta-blocker medication) plus respiratory exchange ratio ≥1.15, volitional exhaustion; or the emergence of concerning symptoms or ECG changes necessitating test termination.

Physical function will be assessed using The Short Physical Performance Battery,^33^ which comprises three components: (a) time to complete five sit-to-stand repetitions, (b) 4 metre gait speed and (c) a series of balance tests. Participants will undergo a further test to count the maximum number of sit-to-stand repetitions (without the use of the arms for support) over a 60 s period (sit-to-stand-60 test).

Muscle strength will be evaluated through several modalities. Quadriceps strength of the right leg will be measured using a fixed dynamometer (BIODEX, Medical Systems, Shirley, New York). Maximal isometric strength will be assessed at 90° knee flexion and defined as the highest peak torque (newton-metre, Nm) across three attempts. Isokinetic strength will be assessed via repeated maximal concentric reciprocal contractions (60°, 90° and 120° per second) for five repetitions. Sets are separated by a 60 s rest.

Handgrip strength will be measured using a hand-held dynamometer, with participants seated, elbow flexed at 90 degrees and the forearm in a neutral position. Each participant will perform three maximal efforts on each hand, alternating sides. The highest force output (kg) from the three attempts on each hand will be recorded for analysis.

#### Cardiometabolic and pathology measures

T2D remission will be assessed at the end of the intervention period (12 weeks±4 weeks) and is defined as achieving an HbA1c level below 6.5% (48mmol/mol) without the reintroduction of any glucose lowering medication. The 12-week timeframe was chosen to align with the active weight-loss phase of an LED currently offered in routine clinical care, during which the greatest absolute and relative reductions in LM typically occur. Delivering the exercise intervention concurrently with this phase will allow for the assessment of LM preservation during negative energy balance, rather than during subsequent weight stabilisation and food reintroduction. Extending the primary endpoint beyond the active weight-loss period could obscure the specific effects of exercise on LM during energy restriction by potentially introducing additional behavioural and metabolic variability.

Venous blood will be collected for the analysis of HbA1c (high-performance liquid chromatography), lipid profile, serum creatinine and liver function and enzyme tests. Urine samples will be analysed for the albumin to creatinine ratio. All biochemical analyses will be conducted in clinical laboratories at the University Hospitals of Leicester, which operate under strict quality control standards and are blinded to treatment allocation.

Blood pressure will be measured using an automated sphygmomanometer following a five min period of seated rest, with systolic and diastolic values averaged across sequential readings. For participants not receiving renin-angiotensin-aldosterone system inhibitors for albuminuria, remission of hypertension will also be assessed at the final visit. Hypertension remission is defined as achieving blood pressure values ≤130/80 mm Hg without the use of antihypertensive medications during the preceding 12 weeks.

#### Resting metabolic rate

Resting metabolic rate (RMR) will be assessed using indirect calorimetry, a validated method for measuring energy expenditure at rest. Participants will be required to attend this assessment in a fasted state, having abstained from food or caloric beverages from 22:00 the night before. RMR will be measured using a ventilated hood system gas exchange measurement indirect calorimeter (GEMNutrition, Cheshire UK), following standardised operating procedures. During the assessment, participants will lie in a supine position and be instructed to remain awake, relaxed and motionless. The ventilated hood will be placed over the head to capture all expired gases over the continuous measurement period (~40 min). The precise quantification of oxygen consumption and carbon dioxide production will be used to calculate RMR.

#### Optional mixed-meal tolerance test

A mixed meal tolerance test will be conducted during a separate visit depending on participant availability, following an overnight fast beginning at 22:00 the previous evening. Participants will consume a liquid mixed meal comprising 250 mL of Fortisip Compact (approximately 600 kcal of which 49% carbohydrate, 16% protein and 35% fat), after a cannula is inserted to allow for repeat blood sampling. Venous arterialised blood samples will be collected to assess parameters of glucose homeostasis, including plasma glucose, insulin and C-peptide concentrations. Samples will be collected in the fasting state (at −60’ and immediately prior to meal ingestion) and at 10’, 20’, 30’, 60’, 90’, 120’, 150’, 180’ postprandially. Questionnaires and visual analogue scales on appetite are completed at each sampling timepoint.^34^

#### Demographic factors, mental well-being and quality of life

Participants will complete a comprehensive questionnaire capturing sociodemographic information, including sex, date of birth, ethnicity, religion, employment status, income, education attainment, marital status, smoking status and alcohol consumption. Psychosocial and health-related quality of life measures are also collected using validated instruments. These include:

1. Hospital Anxiety and Depression Scale^35^ for assessing symptoms of anxiety and depression.
2. Diabetes-related distress (Diabetes Distress Scale-17)^36^ for evaluating diabetes-related emotional burden.
3. EuroQol group 5-Dimensional 5-Level (EQ-5D-5L)^37^ for measuring health-related quality of life.
4. WHO Disability Assessment Schedule (WHODAS V.2.0)^38^ for assessing functional impairment.
5. The Medical Research Council’s Dyspnoea Scale^39^ for evaluating breathlessness severity.

#### Diet, physical activity, sleep and glucose monitoring

At baseline and 12 weeks, participants will undergo comprehensive behavioural and physiological monitoring. To evaluate habitual dietary intake, participants will complete a 4-day standardised diet diary (3 working days and 1 non-working day), which allows for the quantification of total energy intake (including macronutrients and micronutrients) and repeat this at week 12 during the food reintroduction phase.

Participants will wear a blinded continuous glucose monitor (FreeStyle Libre Pro) for up to 14 days at baseline and week 12 (following endpoint assessments). From these data, mean glucose levels, glycaemic variability and time spent within (3.9–10.0 mmol/L), above (>10.1 mmol/L) and below (<3.9 mmol/L) target glucose ranges will be calculated in line with best practice.^40^

Physical activity and sleep will be assessed using a wrist-worn accelerometer (GENEActiv, Activinsights, Kimbolton, UK), worn continuously for 7 consecutive days on the non-dominant wrist at baseline and up to 28 days proximate to starting the intervention and at 12 weeks (following endpoint assessment visit). Accelerometer data will be recorded at 25 Hz and processed through the open-source R programme GGIR^41^ to derive metrics including daily step count, overall acceleration, intensity gradient metric and total time spent at sedentary, light and moderate to vigorous intensities. Overnight wear enables the estimation of sleep duration, total time in bed and sleep efficiency.

### Randomisation and allocation concealment

Following confirmation of eligibility and completion of baseline measures, randomisation will occur at the individual level and be stratified by sex. The randomisation sequence will be developed by an independent statistician and uploaded into a Research Electronic Data Capture (REDCap) system. A researcher blinded to the sequence will unveil group allocation.

### Study interventions

#### Physician review and monitoring

Before intervention initiation, the study physician will consult participants regarding the cessation of glucose-lowering and blood pressure-lowering therapies where applicable.^42^ Glucagon-like peptide 1 receptor agonists and sodium glucose cotransporter 2 inhibitors will be discontinued 1 week before commencing the LED. All other glucose-lowering therapies are stopped on the day of LED initiation. Blood pressure-lowering therapies will be stopped 24 hours prior to LED initiation, except for angiotensin-converting enzyme inhibitors/angiotensin II receptor blockers (ACE I/ARB) prescribed specifically for microalbuminuria or albuminuria or as a third-line therapy. ACE I/ARB may also be continued where three or more blood pressure-lowering therapies are prescribed.

**Figure 1:**
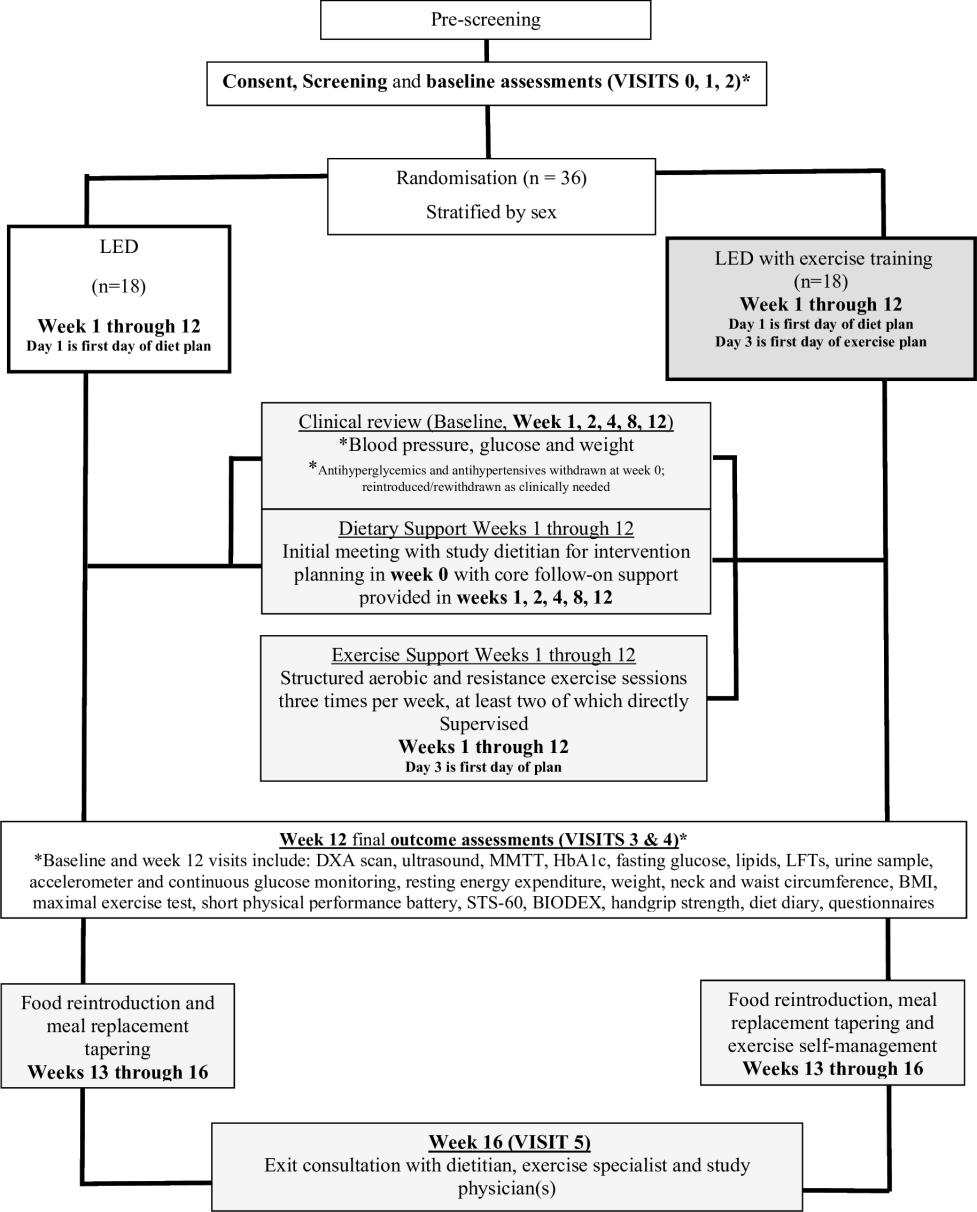
Trial flowchart. BMI, body mass index; DXA, dual energy X-ray absorptiometry; HbA1c, glycated haemoglobin; LED, low energy diet; LFT, liver function test; MMTT, mixed meal tolerance test; STS-60, sit-to-stand 60.

Clinical monitoring will include weekly reviews of 7-point capillary blood glucose profiles, home blood pressure readings (via automated sphygmomanometer) and weight loss readings, at weeks 1, 2, 4, 8, 12 (intervention), 14 and 16 (during food reintroduction) (figure 1).^29^ Reintroduction of medications is guided by safety criteria; glucose-lowering therapies are reinstated only if fasting blood glucose exceeds 10 mmol/L in >50% of readings, subject to physician discretion and participant discussion. Blood pressure medications are reintroduced if systolic blood pressure exceeds 165 mm Hg during weeks 1 and 2, or 150 mm Hg from week 3 onwards.^43^ Participants with childbearing potential will be required to use reliable methods of contraception, discussed prior to randomisation.

#### Diet-only group (group 1)

Participants will follow a 12-week MRP-based LED using products from Counterweight and Optifast (Nestlé), providing 800–900 kcal/day (approximately 35% protein, 50% carbohydrate and 15% fat). To prevent constipation, participants are advised to consume ≥2 L of calorie-free fluid daily, include two portions of non-starchy vegetables and use a fibre-based laxative (Optifibre) as needed.

Adequate protein intake in this trial is a separate and important consideration, as populations living with T2D typically under-consume protein in relation to recommendations.^44^ Adequate protein intake during weight loss may act to both augment the LM-preserving effect of exercise and to independently aid fat reduction (through the thermic effect of feeding) and LM preservation.^45^,^47^ Participants will consume a minimum of 80–100 g of protein per day through the LED, which will provide approximately 1 g/kg/day of protein. While additional protein by itself has not been found to aid LM preservation in adults living with obesity undertaking an LED,^48^ this has not been studied in combination with a structured supervised exercise intervention or in a South Asian population.

Dietitian consultations will occur at weeks 1, 2, 4, 8 and 12 of the intervention (figure 1), with additional sessions available on request to support the MRP-based LED. Consultations may be delivered in-person or virtually, with in-person consultations allowing monitoring of body weight, blood glucose and blood pressure. Monitoring of self-reported diet adherence will be complemented through the distribution and return of MRPs.

Following the intervention, participants will collaborate with the dietitian to develop a personalised food reintroduction plan over a minimum of 4 weeks. This plan aims to support weight maintenance, optimise glycaemic control and ensure nutritional adequacy in line with national dietary guidelines.

#### Diet and exercise group (group 2)

Participants in this group will follow the same dietary protocol as group 1, with energy intake adjusted on exercise days via an additional MRP or an energy and protein equivalent meal. Beginning on day 4 of the diet, participants engage in a structured exercise programme supervised by an exercise physiologist at the Leicester Diabetes Centre.

The exercise programme includes two supervised sessions per week combining resistance and aerobic training and one home-based aerobic session. Compliance with home-based exercise is assessed during supervised visits. The goal is to achieve up to 3 60 min sessions per week.

Resistance training includes 1–3 sets of 8–12 repetitions across four upper body exercises (bench press, seated row, shoulder press and pull down), three leg exercises (leg press, extension and flexion) and functional movements targeting stability and posture. Resistance is increased once 12 repetitions are completed with proper technique. Resistance bands are provided for continued use for home-based resistance exercise.

Aerobic exercise consists of brisk treadmill walking at 60%–80% of peak heart rate from baseline stress testing. For participants with contraindications to walking (eg, plantar fasciitis), a cycle ergometer will be offered during supervised exercise and an arm ergometer provided for home-based exercise sessions. Training intensity will be progressively modified to ensure an adequate physiological stimulus is maintained throughout the intervention. Participants will gradually increase session duration, aiming to achieve 60 min sessions over time.

#### End of intervention

At the conclusion of the intervention, participants will meet with both the dietitian and the exercise physiologist to discuss strategies for maintaining weight loss and physical activity. The study physician will provide recommendations for long-term medication management to the participant’s general practitioner, based on HbA1c and blood pressure outcomes, in accordance with national diabetes care guidelines.

### Monitoring for adverse events

Intervention team members will enquire about adverse events at each visit. Participants will be asked to contact trial staff between visits to report any concerns. Serious adverse events will be reported within 24 hours to sponsor, which may seek a formal adjudication of relatedness by physicians blinded to the treatment arm and who have not interacted with the study participants. Common adverse events in LED trials^10 49^ include constipation (18%–47%), headache (8%), dizziness (4%–32%), fatigue (11%–25%) and thirst (6%). Constipation responds to fibre-containing laxatives and fluid intake, and other symptoms resolve over time. In one high-intensity aerobic training trial,^50^ 16% experienced fracture or lower extremity muscle cramping and/or muscle, ligament or tendon strain; 5.2% experienced chest pain, difficulty breathing, dizziness or loss of consciousness. In the LITOE trial’s combined resistance and aerobic exercise arm,^22^ 12.5% had one of shoulder injury, left knee pain, spinal stenosis exacerbation or hip pain; one participant in the resistance arm experienced atrial fibrillation.

### Withdrawal

Participants have the right to withdraw from the study at any time without needing to provide a reason. If a participant does withdraw, efforts will be made to understand the reason for withdrawal, for a better understanding of the experience of the intervention arm. Participants may be withdrawn from the study interventions at any time due to (for example) intolerance to the study intervention, significant non-compliance, pregnancy, prolonged or serious hospital admission or development of active malignancy or serious illness.

### Sample size

Based on published data,^51^ 32 participants (16 completers in each intervention arm) will be required to detect a 1 kg difference in LM assessed via DXA at study endpoint between intervention arms with 80% power, two-sided 0.05 significance level and an assumed SD of ±1 kg LM.^51^ Allowing for up to a 10% dropout rate, 36 participants (18 in each arm) are needed. The low drop-out is based on our ongoing RESET for Remission trial, currently offering a LED (of which >50% recruited are of South Asian ethnicity), which currently has 7.5% drop-out (UK site).^29^

### Data management

Data management is facilitated by institutional REDCap systems, with access controlled through Active Directory Technology. Plausible ranges for all outcome measures have been derived within REDCap; values outside these ranges are automatically flagged and verified for accuracy.

### Statistical analyses

The full statistical analysis plan can be found in the Supplementary Material (online supplemental file 2). Participants with complete data will be analysed in the groups to which they were randomised (ie, using modified intention-to-treat principles). Every effort will be made to obtain the primary outcome. The primary outcome is a continuous variable and will be analysed using linear regression modelling. Change between baseline and follow-up will be included as the dependent variable, with randomisation group added as an independent binary factor (diet, diet and exercise). Models will be adjusted for baseline values and randomisation stratification factor (sex). Data distribution will be assessed for normality, with an appropriate link function (providing the best model fit) selected if the data are not normally distributed. Data will be reported as within-group change (mean, 95% CI), along with the mean (95% CI) intervention effect (change in exercise and diet intervention minus change in diet intervention arm).

Secondary outcomes will be analysed using the same modelling approach as the primary outcome. Categorical secondary outcomes will be analysed using Fisher’s exact test statistics. Significance levels will not be adjusted for the multiple outcomes but will be interpreted in relation to the primary outcome and overall pattern of results, acknowledging that secondary outcomes may be underpowered. Missing baseline data will be replaced and included using the indicator method so cases are not dropped where follow-up data is present without baseline data.

A sensitivity analysis will be undertaken for the primary outcome that analyses all randomised participants with missing data, replaced with multiple imputation. A per-protocol analysis will also be undertaken for the primary outcome, restricting the intervention group to those who achieved at least 5% weight loss and adhered to at least two-thirds of prescribed exercise sessions.

### Trial oversight and governance

The study is sponsored by the University of Leicester (RGOsponsor@leicester.ac.uk), who will ensure the study is conducted according to Good Clinical Practice and that all contractual, governance, ethics and regulatory processes are followed. A trial management group meets at least monthly. This group includes the Chief Investigator and Co-Investigators (as appropriate), and the day-to-day project management team to provide appropriate oversight of the trial. A data monitoring committee was not judged to be needed by the sponsor.

### Trial status

The latest approved protocol is V.2.3 (12.06.2025). This trial is in follow-up. The statistical analysis plan will be uploaded and accessible on the International Standard Randomised Controlled Trial Number Registry (ISRCTN11175684).

### Patient and public involvement

Local patient support groups supported the development of the intervention and provided input into the acceptability of the intervention and the proposed study assessment visits. Participants who have expressed an interest in receiving a summary of the study findings will be informed of the results at the end of the study.

## Discussion

Intentional attempts to reduce excess adiposity for the treatment of obesity and remission of T2D frequently result in unintended loss of LM, particularly skeletal muscle, which plays a critical role in both physiological and metabolic health. To date, the extent to which these LM losses can be mitigated in South Asian adults, who typically present with lower LM and higher central and ectopic adiposity,^15 19^ has not been studied.

The STANDBy feasibility trial^52^ recently evaluated the acceptability of MRP-based LED (providing approximately 0.6 g/kg/day of protein) among South Asian adults living with T2D, aged 18–65 years. The trial demonstrated comparable remission outcomes to the DiRECT study, despite a lower overall percentage of weight loss (6.5% vs 8.8% in DiRECT) and confirmed significant reductions in LM volume assessed by MRI. This trial, however, had a small sample size (n=25) with a notable COVID-19-related disruption, which limited the strength of conclusions. A subanalysis of the DIASTOLIC trial^53^ found that South Asian adults living with T2D and obesity undertaking an MRP-based LED experienced a higher drop-out (4 of 12 vs 0 of 15 in White Europeans) rate, lost less visceral adipose tissue and had smaller improvement in markers of insulin resistance compared with White Europeans.^54^ Notably, both trials did not incorporate a concurrent exercise intervention.

In contrast, the DIADEM-I trial^49^ conducted in Qatar recruited younger adults (18–50 years) with a recent T2D diagnosis (≤3 years) from Middle Eastern and North African backgrounds. Participants were randomised to receive either an LED (providing approximately 0.5/g kg/day of protein) with physical activity support during the food reintroduction phase or standard care. The intervention group demonstrated greater fat mass reduction (−9.97±9.06 kg vs −2.89±6.41 kg), while fat-free mass losses were similar between groups (−1.41 kg±2.92 vs −1.33 kg±2.94), suggesting physical activity may have helped to preserve LM and improve overall body composition. However, LM was assessed using bioimpedance, which has known limitations in individuals with obesity,^55^ and the exercise component was introduced postweight loss, supporting muscle regain after weight loss rather than preservation during weight loss.

Understanding whether structured exercise can enhance health outcomes during intensive dietary interventions is essential for informing clinical practice guidelines. Preserving resting energy expenditure through LM maintenance may also help prevent weight regain. Given that LM is metabolically active,^56^ demanding approximately 13kcal/kg/day and is not typically regained to the same extent as fat mass following weight regain,^57^ its preservation is important not only for metabolic health but also for sustaining physical function and reducing long-term risks such as frailty and loss of independence.^58^

This trial represents a significant step forward in tailoring treatments for individuals with T2D, particularly among South Asian populations who experience disproportionately higher rates of T2D and other multiple long-term conditions (multimorbidity). By focusing on this understudied population, this study offers a valuable opportunity to explore their unique physiological and metabolic responses and to help personalise future treatment options. The objective measures of physical activity and glycaemic control, supplemented with diet diary self-report and quality of life, will enable a comprehensive characterisation of the 24-hour lifestyle behaviours of this sample and describe how these behaviours and glycaemic profile change following a programme of intensive weight loss and cessation of glucose-lowering medications. This will be complemented by the assessment of dropout rates and adherence to better understand the acceptability of this intensive approach, beyond its potential efficacy in improving health outcomes. Optimising physical function, promoting T2D remission and preserving LM through combined resistance and aerobic exercise during an LED may offer a comprehensive strategy. This approach could safeguard against the long-term health risks associated with LM loss, enhance body composition and reduce the risk of frailty, ultimately supporting greater independence and quality of life in this high-risk population.

This study has several limitations that should be considered. The open-label design may allow changes in participant behaviour that are not fully attributable to the prescribed interventions, and differential contact between groups, introduced through supervised exercise sessions, may introduce bias. These risks are partially mitigated through the use of objective, validated outcome measures and blinded assessment of primary endpoints and laboratory analyses. It is also possible that participants allocated to the LED-only group independently alter physical activity behaviours during the intervention period; however, objectively measured accelerometer data are collected to characterise habitual activity and monitor changes over time. In addition, the single-centre setting, relatively short intervention duration and limited sample size may limit the generalisability of findings, preclude evaluation of longer term behavioural or physiological maintenance and lead to type 2 errors in secondary outcomes.

## Ethics and dissemination

The trial was approved by the NHS research ethics service (23/WM/0201). The study is conducted in accordance with the Declaration of Helsinki 2024 and adhered to the regulations for Good Clinical Practice.

The results of the study will be published in relevant medical journals and disseminated at national and international conferences and meetings. Participants will also be informed about the results of the study. Acknowledgement of the role of any supporting organisations, including Wellcome Trust, and the University of Leicester, will be included. Data request processes will be developed for investigators, collaborators and their teams to conduct follow-up data analyses once the primary paper has been published using anonymised data.

## Supplementary material

10.1136/bmjopen-2025-110459 online supplemental file 1

10.1136/bmjopen-2025-110459 online supplemental file 2

## Data Availability

Data are available upon reasonable request.
