# Supplementary material for: Combining structured exercise with a low-energy diet to attenuate lean mass loss in South Asian adults living with type 2 diabetes: the COMBINE randomised trial protocol
Source: BMJ Open. 2026 Mar 23;16(3):e110459. doi: 10.1136/bmjopen-2025-110459 (PMC13034260; doi:10.1136/bmjopen-2025-110459)
Supplement: online supplemental file 1 [file bmjopen-16-3-s001.pdf]

Sponsor: University of Leicester

Participant Screening ID Number: ______________________

CONSENT FORM

Title of Project: combining structured exercise with a high protein low energy diet to attenuate lean mass loss in South Asian adults living with type 2 diabetes: the COMBINE trial

Chief Investigator: Professor Thomas Yates

Please **initial** all boxes

1	I confirm that I have read and understood the information sheet dated 26/03/2024 (version 3.1) for the above study. I have had the opportunity to consider the information, ask questions and have had these answered satisfactorily.		
2	I understand that my participation is voluntary and that I am free to withdraw at any time, without giving any reason and without my medical care or legal rights being affected. My data and samples collected up to that point will still be used for research purposes now and in the future.		
3	I understand that relevant sections of my medical notes and data collected during the study may be looked at by individuals from the study team, the University of Leicester, from regulatory authorities or from the host NHS Trust, where it is relevant to my taking part in this research. I give permission for these individuals to have access to my records.		
4	I understand that my General Practitioner and/or other healthcare professionals will be informed of my participation in this study, and of my study results. I understand that my GP will be contacted to provide information about my health status to the research team for confirmation of eligibility.		
5	FEMALE PARTICIPANTS ONLY: I understand I will be withdrawn from the study if I become pregnant<br>MALE PARTICIPANTS ONLY: Please circle "N/A" (Not Applicable)		N/A
6	I agree to take part in the above study.		
	**The following statements are OPTIONAL – please initial either Yes or No to each of the following statements.**	YES	NO
7	I wish to receive a copy of the study results, and and I agree for my contact details to be retained and used for this purpose.		
8	I understand that my coded research data and samples may be shared with other researchers, organisations, collaborators and commercial partners (or their delegate) both inside and outside of the UK.		

9	I give permission for my blood samples which remain after the end of the study to be stored for use in future ethically approved studies. NB. where samples are retained and/or transferred to a different organisation, we will also retain/supply a copy of your signed consent form to the custodian of the samples.		
10	I agree to being contacted with details of future relevant research and to details being stored on the Leicester Diabetes Centre Volunteer database for the purpose of future research.		
11	I agree that my information held by the NHS and records maintained by the General Register Office may be used to follow-up my health status for up to 10 years even if I do not attend future study visits.		

Name of Participant	Date	Signature

Name of Person receiving consent	Date	Signature
