# Supplementary material for: Combining structured exercise with a low-energy diet to attenuate lean mass loss in South Asian adults living with type 2 diabetes: the COMBINE randomised trial protocol
Source: BMJ Open. 2026 Mar 23;16(3):e110459. doi: 10.1136/bmjopen-2025-110459 (PMC13034260; doi:10.1136/bmjopen-2025-110459)
Supplement: online supplemental file 2 [file bmjopen-16-3-s002.pdf]

Statistical Analysis Plan

Combining structured exercise with a low energy diet to attenuate lean mass loss in South Asian adults living with type 2 diabetes: THE COMBINE TRIAL

Trial registration number: ISRCTN11175684

SAP revision history

Date	Version	Justification for SAP version
25 July 2025	0.1	First draft for investigator review
28 August 2025	0.2	Second draft for investigator review
29 August 2025	1.0	Approved SAP

SAP responsibilities

Role in SAP development	Name, affiliation	Role in trial
SAP author 1	Franciskos Arsenyadis, University of Leicester	Study Co-Investigator
SAP author 2	Thomas Yates, University of Leicester	Study Chief Investigator
SAP author 3	Dr Maryam Yazdi	Trial Statistician
SAP author 4	Katerina Tziannou	Trial Statistician
SAP reviewer 4		
SAP reviewer 5		

SAP signatures

Role	Name, affiliation	Date	Signature
Co-Investigator	Franciskos Arsenyadis<br>Leicester Diabetes Centre<br>University of Leicester	27.01.2026	F. Arsenyadis
Chief Investigator	Prof Tom Yates<br>Leicester Diabetes Centre<br>University of Leicester	27.01.2026	T. Yates

1 Introduction

1.1 Trial background and rationale

Type 2 diabetes (T2D) occurs earlier and more frequently in South Asians living in the UK, with implications for longer term morbidity and mortality. Low energy diets (LED) incorporating meal replacement products can induce diabetes remission through weight loss, but often also cause losses to lean body mass (LBM). Combining resistance and aerobic exercise with LED could help promote LBM preservation, as well as enhance markers of physical function and strength, but this has not been examined. The COMBINE trial investigates the effect of exercise and LED on LBM preservation, compared to LED alone, with markers of physical function and strength as key secondary outcomes.

1.2 Primary trial objectives

1.3 To investigate whether combining structured exercise with a low energy diet (LED) leads to amelioration of lean body mass (LBM) loss (measured by Dual Energy X-ray Absorptiometry [DXA]) in South Asian adults aged between 40 and 65 years living with T2D and obesity compared to a LED alone

1.4 Key secondary trial objectives

To investigate whether a low energy diet combined with exercise training in this population results in the following outcomes, compared to low energy diet alone, at 12 weeks:

- Improvements in other measures of body composition (e.g body fat percentage reduction)
- Improved markers of muscle strength, physical function, and cardiorespiratory fitness
- Improvements in other clinical markers of cardio-metabolic-renal and liver health

We list the specific outcomes in Table 1, with additional indication as to whether the variables are categorical or continuous.

**Table 1 Outcomes**	**Week 12**	**Continuous**	**Categorical**
**Primary outcome**			
Lean body mass (DXA)	**X**	**X**	
**Key secondary outcomes**			
DXA-derived body composition measures			
Appendicular lean mass (arms, legs and total)	**X**	**X**	
Total and percentage fat mass	**X**	**X**	
Visceral adipose tissue	**X**	**X**	
Total bone mineral density and bone mineral content	**X**	**X**	
Regional bone mineral density (hip, femoral neck	**X**	**X**	
Other anthropometric assessments			
Weight and Body Mass Index	**X**	**X**	
Waist and neck circumference	**X**	**X**	
Indirect calorimetry			
Resting metabolic rate	**X**	**X**	
Muscle ultrasound			
Quadriceps muscle diameter, volume and quality	**X**	**X**	
Main fitness measure			
VO_2 peak (absolute, relative to lean body mass and total body mass)	**X**	**X**	
Other exercise stress test measures			
VCO_2 peak, maximum gradient achieved	**X**	**X**	
Physical function and strength			
Handgrip strength	**X**	**X**	
Isometric and isokinetic quadriceps strength (Biodex) (absolute and relative to lean body mass and total body mass)	**X**	**X**	
Short Physical Performance Battery	**X**	**X**	
Sit-to-stand 60	**X**	**X**	
**Other secondary outcomes**			

Cardiometabolic and pathology measures			
Diabetes remission at 12 weeks	**X**		**X**
Haemoglobin A1c	**X**	**X**	
Systolic and diastolic blood pressure, heart rate	**X**	**X**	
Hypertension remission	**X**		**X**
Renal function measures			
Creatinine and estimated glomerular filtration rate	**X**	**X**	
Urine albumin to creatinine ratio	**X**	**X**	
Hepatic function measures			
Alkaline phosphatase, alanine transaminase, aspartate transaminase, gamma-glutamyl-transferase	**X**	**X**	
Total cholesterol, HDL, LDL, triglycerides	**X**	**X**	
Continuous glucose monitoring			
Mean glucose levels, variability and time within, above and below range		**X**	
Optional mixed-meal tolerance test			
Fasting and post-prandial insulin and glucose levels	**X**	**X**	
Overall health state			
EuroQuol group 5-Dimensional 5-Level Questionnaire	**X**	**X**	
WHO Disability Assessment Schedule 2.0	**X**	**X**	
Dyspnoea scale	**X**		**X**
Depression, anxiety and distress			
Hospital Anxiety and Depression Scale and Diabetes Distress Scale	**X**	**X**	
**Process outcomes related to the intervention**			
Dietary variables			
Total energy and macronutrient intake (protein, carbohydrates, lipids)	**X**	**X**	
Selected carbohydrate (total sugars, fibre) and lipid (saturated, monounsaturated, polyunsaturated) types, relevant micronutrients and alcohol intake	**X**	**X**	
Accelerometer-based physical activity measures and sleep (daily average)			
Steps, overall acceleration, and intensity gradient metric	**X**	**X**	
Minutes for each of sedentary, light and moderate to vigorous physical activity	**X**	**X**	
Sleep time, duration of night, sleep efficiency (sleep time/duration of night)	**X**	**X**	
Intervention acceptability			
Completion rates and percentage weight loss achieved	**X**	**X**	**X**

Table 1. Trial outcomes and variable types

2 Methods

2.1 Trial design

The trial is a single centre parallel group randomised controlled trial, in which participants are randomised to either a low energy diet intervention (diet group) or a low energy diet combined with exercise training (diet and exercise group). Participants are followed up for 12 weeks (concurrent with interventions).

2.2 Randomisation

Individual randomisation (1:1) is stratified by sex (female/male) in blocks of variable size.

2.3 Blinding

Group allocation is open to both the participant, treating physician and the rest of the intervention delivery team. However, it is a blinded end-point trial, meaning the batch analysis of LBM changes which is the primary outcome, and the trial statistical analyst team will be blinded to allocation.

2.4 Sample size

In order to achieve the sample size calculation specified in the trial protocol, we estimated that 32 individuals would be required to complete the trial. This will enable detection of a 1kg difference in LBM assessed by DXA at 12 weeks between interventions arms with 80% power, two-sided 0.05 significance level and assumed standard deviation of ± 1kg LBM [1].

2.4 Framework

This is an efficacy trial. We will compare the primary and secondary outcomes in the diet + exercise group to the diet group.

2.5 Interim analyses and stopping guidance

None planned.

2.5 Timing of final analysis

The Trial Statistician at Leicester Diabetes Centre will perform analyses described in this SAP following completion of the trial and database lock.

2.6 Timing of outcome assessments

We assess outcomes at 12 ± 4 weeks– see **Table 1**.

3 Statistical principles

3.1 Confidence intervals

We will report estimates of effect with 95% confidence intervals.

3.2 Intervention adherence

Adherence to the dietary component of the intervention will be summarised as follows:
The number (%) achieving a weight loss threshold of at least 5% at 12 weeks will be considered as adherent diet responders.

Adherence to the exercise component of the intervention will be summarised as follows:
The number (%) of prescribed and supervised exercise sessions completed will be reported. Attendance of at least two thirds of supervised or home-based sessions will be considered as adherent.

3.3 Analysis population

The primary outcome (LBM change at 12 weeks) and the secondary outcomes will use a modified Intention-to-Treat (ITT) approach, in which individuals with complete data are included in the group to which they were randomised. A sensitivity analysis will assess the impact of missing data using multiple imputation (see **section 5.2.4**).

All secondary outcomes will also use a modified ITT population whereby individuals are included in the group to which they were randomised using a complete case sample (i.e. those with missing follow-up data will be removed from the analysis).

A sensitivity analysis of the primary outcome will be undertaken using a Per-Protocol (PP) population, comprising the following:

Diet intervention – all individuals who have adhered to the diet component of the intervention

Exercise + diet intervention – those judged to have adhered to both the diet and exercise components of the intervention (see **section 3.2** for definitions)

4 Trial population

4.1 Eligibility criteria

Eligibility criteria for the COMBINE trial are summarised below:

**Table 2 Eligibility criteria**	
**Inclusion criteria**	
Ethnicity	• South Asian ethnicity (Self-declared ethnicity of self)
Age	• Aged ≥40 and ≤ 65 years
Diabetes status	• Clinically coded diagnosis of T2D between 3 months and 10 years previously
Glycaemic control	• HbA1c 6.5% (48 mmol/mol) to 10% (86 mmol/mol) if not taking glucose-lowering medication; 6% (42 mmol/mol) to 10% (86 mmol/mol) if taking glucose-lowering medication
Diabetes medication(s)	• Treatment stable; no significant change to glucose-lowering regimen in the preceding 3 months, as determined by a study investigator
Body Mass Index	• ≥ 25 and ≤ 45 kg/m^2
Weight trajectory	• Self-reported stable weight over the previous 6 months (<± 5% of bodyweight)
Consent	• Able to provide informed consent<br>• Able to understand written and spoken English or willing to use the University Hospitals of Leicester professional interpreter service<br>• Able to take part in structured exercise training requiring the lower limbs (e.g., able to walk without assists or impairment)
Willingness	• Willingness and availability to participate in the proposed interventions to which they may be assigned, including attendance of intervention visits such as exercise sessions and adoption of LED which requires abstinence from alcohol<br>• Willingness to self-monitor glucose, blood pressure and weight
**Exclusion criteria**	
Diabetes status	• Individuals with type 1, gestational or monogenic diabetes mellitus

Insulin use	• On insulin therapy (NOTE: No COMBINE participant will be on insulin at baseline as this is an exclusion criterion. An exception may made for women who are on insulin because of its lack of teratogenic effects rather than because of inability to control glycaemia on oral agents alone)<br>• On oral or injected steroids<br>• On weight loss medications (not including glucose lowering medication)
Kidney function	• eGFR <45 $ml.min^{-1}$ per $1.73m^2$
Surgical history	• Previous bariatric surgery
Eating disorder	• Self-reported or diagnosed eating disorder
Diet	• Self-reported milk protein allergy or other allergy or dietary practice that prohibits the use of meal replacement products
Cardiovascular health	• Previous myocardial infarction, stroke, amputation secondary to T2D/peripheral vascular disease, or admission due to CVD-related event within 12 months<br>• Previous clinically diagnosed atrial fibrillation<br>• Previous clinically diagnosed heart failure<br>• Pacemaker or implantable cardioverter defibrillator (ICD)<br>• Presenting with cardiac abnormalities during the exercise ECG test (inclusive of very high blood pressure)
Eye health	• Currently receiving or requiring active treatment for retinopathy.
MMTT	• Severe Intolerance or unwillingness/inability to undertake Mixed Meal Tolerance Test (Severity of intolerance to be assessed by a member of the research team during screening visit)
Other	• Current participation in another research study with investigational medical product<br>• Currently participating in a weight reduction program in addition to routine care<br>• Conditions that could impact weight (i.e., active malignancy/treatment in past year, pregnancy, lactation, planning to become pregnant in next 8 months)<br>• Drugs or conditions thought by the investigators to have significant impact on the study protocol or outcomes<br>• Substance abuse. The requirement for alcohol abstinence during the initial 12 weeks will make it unlikely that individuals with alcohol dependence will enrol. Substance abuse will be queried.

4.2 Recruitment and screening

We will present a CONSORT diagram outlining participant flow throughout the study. This will include the number of individuals invited to participate; categorized by source (e.g., primary care, secondary care, existing databases, publicity or press releases, and participant referrals), as well as the number reached, screened for eligibility, and deemed eligible. We will also report the number of individuals who expressed interest or declined participation (including reasons for non-participation when available), those who

completed the screening calls, or baseline visits, and those who were randomized. Finally, the diagram will capture the number of participants who were withdrawn or lost to follow-up, who completed trial evaluations, and who adhered to trial procedures.

4.3 Baseline characteristics

The following baseline characteristics will be summarised by randomised group in Table 1, using mean (SD) or median (IQR) as appropriate for continuous variables, and number and percentage for binary or categorical variables.

- Age (yrs).
- Sex (female/male)
- Ethnicity (Indian, Pakistani, Bangladeshi or Other Asian)
- Religion
- Social deprivation through Index of Multiple Deprivation (IMD) score
- Family history of diabetes in first-degree relatives (yes/no).
- Diabetes Duration
- Medication (glucose lowering, antihypertensive, lipid lowering).
- Smoking status (current, past, never)
- Alcohol intake (never, ex-drinker, current drinker)
- Employment type (full time, part time, unemployed, retired, other)

Baseline values of all reported secondary outcome variables will be incorporated into the Table of Results.

5 Analysis

5.1 Outcomes

5.1.1 Primary outcome

The primary outcome is change in LBM at 12 weeks as measured by DXA scan; a continuous outcome measured in kilograms.

5.1.2 Secondary outcomes

We report secondary outcomes and follow-up time points in **Table 1.**

5.2 Analysis methods

5.2.1 Analysis of the primary outcome

The mean and SD of LBM will be calculated at baseline, by randomised group. Mean and SD of the change values will be reported between baseline and 12 weeks. Data will be reported as within group change (mean, 95% CI), along with the mean (95% CI) intervention effect (change in exercise + diet intervention minus change in diet intervention arm).

For the primary outcome, the intervention effect, confidence interval and p-values for the comparison between intervention groups will be derived from a linear regression model with change in LBM between baseline and follow up as the dependant variable and randomisation group included as an independent binary fixed factor (diet, diet and exercise). Baseline LBM value, randomisation stratification variable (sex) and age will be included as covariates. It is anticipated that the primary outcome will be normally distributed. This will be inspected and alternative tests will be considered where data are not normally distributed. $P<0.05$ will be considered significant.

We will repeat these analyses for the per-protocol population (see **section 4.2**)

5.2.2 Analysis of secondary and tertiary outcomes

Secondary outcomes will be analysed using the same modelling approach as the primary outcome.

We will analyse the categorical secondary outcomes (e.g diabetes remission at 12 weeks, hypertension remission at 12 weeks) using Fisher's exact test statistics. We define T2D remission as an HbA1c of < 6.5% (48 mmol/mol) at 12 weeks without the use of glucose lowering medication during prior 12 weeks. We define hypertension remission as a systolic/diastolic blood pressure of ≤ 130/80 mm Hg without use of antihypertensive agents during prior 12 weeks.

5.2.3 Subgroup analyses for primary outcome

The primary outcome model will be repeated within (stratified) by the following subgroups: degree of weight loss (<10%, ≥10%), and for sex.

5.2.4 Missing data

Primary outcome and categorical secondary outcomes at 12 weeks

For the primary outcome, an additional sensitivity analysis will be undertaken by replacing missing data with multiple imputation by chained equations, with 10 imputed datasets. Variables including treatment arm, age, sex, baseline BMI and baseline outcome value will be included in the imputation model, in addition to auxiliary variables which may be associated with missingness.

All continuous secondary/ tertiary outcomes: missing follow-up data

For those with missing follow-up data, information from clinical records will be extracted where available for appropriate secondary outcomes (if they have been obtained +/- 6 weeks of the 12-week follow-up date. For all other continuous secondary outcomes, we will exclude participants with missing data at 12 weeks. This complete-case analysis is valid under the assumption that the outcome is missing at random (MAR), conditional on randomised group, baseline value and other covariates in the model. For categorical diabetes and hypertension remission variables, missing data will be assumed as no remission.

5.2.5 Multiplicity

We will not make any formal corrections to account for the number of secondary outcomes. However, all secondary outcomes listed as 'other secondary outcomes' in Table 1 will not be interpreted in isolation but in relation to the overall pattern of results.

5.3 Safety data

We will summarize the number (%) of individuals experiencing a serious adverse event, or any adverse event deemed as related to the dietary or exercise intervention, by randomised group.

5.4 Statistical software

SPSS v29 or RStudio v2025.05.01 (or latest available versions).

6 References

1. Longland TM, Oikawa SY, Mitchell CJ, Devries MC, Phillips SM. Higher compared with lower dietary protein during an energy deficit combined with intense exercise promotes greater lean mass gain and fat mass loss: a randomized trial. Am J Clin Nutr. 2016 Mar;103(3):738–46.

2. White IR, Thompson SG. Adjusting for partially missing baseline measurements in randomized trials. Statist. Med. 2005;24:993-1007.
