# Supplementary material for: Combining structured exercise with a low-energy diet to attenuate lean mass loss in South Asian adults living with type 2 diabetes: the COMBINE randomised trial protocol
Source: BMJ Open. 2026 Mar 23;16(3):e110459. doi: 10.1136/bmjopen-2025-110459 (PMC13034260; doi:10.1136/bmjopen-2025-110459)
Supplement: Supplementary file 3 [file bmjopen-2025-110459.reviewer_comments.pdf]

PEER REVIEW HISTORY

BMJ Open publishes all reviews undertaken for accepted manuscripts. Reviewers are asked to complete a checklist review form and are provided with free text boxes to elaborate on their assessment. These free text comments are reproduced below.

ARTICLE DETAILS

Title (Provisional)

Combining structured exercise with a low-energy diet to attenuate lean mass loss in South Asian adults living with type 2 diabetes: The COMBINE randomised trial protocol

Authors

Arsenyadis, Franciskos; Henson, Joseph; McCarthy, Matthew; Papamargaritis, Dimitris; King, James A; Redman, Emma; Whelehan, Gráinne; Wilkinson, Thomas; Sargeant, Jack; Rowlands, Alex; Boulé , Normand; Dasgupta, Kaberi; McCann, Gerry P; Davies, Melanie J; Khunti, Kamlesh; Choudhary, Pratik; Yates, Thomas

---

VERSION 1 - REVIEW

**Reviewer**	**1**
**Name**	**Cao, Yingting**
**Affiliation**	**La Trobe University**
**Date**	**29-Dec-2025**
**COI**	**None**

Thank you for submitting this well-designed and clinically meaningful protocol. The study addresses an important evidence gap by examining whether structured exercise can mitigate lean mass loss during intensive low-energy diet–induced weight loss in South Asian adults with type 2 diabetes. The rationale is strong, methods are generally robust, governance and ethics are well described, outcomes are comprehensive, and transparency in registration, funding, and conflicts is excellent. Overall, this protocol is of high quality and suitable for publication following clarification of key areas:

1. BMI threshold- Please justify the choice of BMI ≥25 kg/m², particularly given South Asian–specific cut-offs and your rationale that diabetes develops at lower BMI in this population. Clarification of scientific, feasibility, and generalisability implications is needed.

2. primary timeframe (12 weeks)- Provide clearer justification for selecting a 12-week endpoint for lean mass preservation and acknowledge whether this reflects a clinically meaningful and physiologically appropriate period.

3. Explicitly acknowledge the potential bias introduced by the open-label design and greater structured contact in the exercise arm, and clarify any strategies to mitigate or monitor this.

4. Please clarify how ethnicity is determined and whether subgroup analyses or contextual considerations (e.g., nationality differences) will be explored.

5. The analysis plan is broadly appropriate but would benefit from brief clarification regarding handling of missing data (particularly given small N and modified ITT), limitations of multiple imputation, and acknowledgement that secondary outcomes are exploratory and underpowered.

VERSION 1 - AUTHOR RESPONSE

Response to Dr Cao:

Thank you for submitting this well-designed and clinically meaningful protocol. The study addresses an important evidence gap by examining whether structured exercise can mitigate lean mass loss during intensive low-energy diet–induced weight loss in South Asian adults with type 2 diabetes. The rationale is strong, methods are generally robust, governance and ethics are well described, outcomes are comprehensive, and transparency in registration, funding, and conflicts is excellent. Overall, this protocol is of high quality and suitable for publication following clarification of key areas:
1. BMI threshold- Please justify the choice of BMI ≥25 kg/m², particularly given South Asian–specific cut-offs and your rationale that diabetes develops at lower BMI in this population. Clarification of scientific, feasibility, and generalisability implications is needed.

Response:

We thank the reviewer for this important comment. Although type 2 diabetes commonly develops at lower BMI in South Asian populations, the use of intensive weight loss interventions at lower BMI thresholds remains an area of ongoing debate. For this efficacy-focused physiological trial, a BMI threshold of ≥25 kg/m² was selected to ensure participants had sufficient excess adiposity to safely undergo a low energy diet and to minimise heterogeneity in diabetes phenotype, body composition and metabolic risk. This threshold also reflects current clinical eligibility criteria for attempting low energy diet–based diabetes remission interventions in routine practice. This clarification has been added to the Eligibility criteria section.

From a scientific perspective, inclusion of individuals at lower BMI could introduce greater variability in lean mass, fat distribution, physical function and underlying pathophysiology, which may obscure the specific effects of concurrent exercise on lean mass preservation during energy restriction. From a feasibility standpoint, recruitment of individuals with BMI ≥25 kg/m² reflects the most common clinical presentation of type 2 diabetes within the local South Asian population and aligns with existing weight-management services.

We do however acknowledge that emerging evidence suggests modified intensive dietary interventions may also be effective at lower BMI in selected individuals (1); however, such populations may represent distinct clinical phenotypes and low energy diets are not currently offered at these BMI thresholds in routine care. The chosen BMI range therefore represents a

pragmatic balance between physiological homogeneity, intervention safety, feasibility, and generalisability to routine clinical practice.

2. primary timeframe (12 weeks)- Provide clearer justification for selecting a 12-week endpoint for lean mass preservation and acknowledge whether this reflects a clinically meaningful and physiologically appropriate period.

The 12-week timeframe was chosen to align with the active weight-loss phase of a low energy diet, actively being offered in routine clinical care, during which the greatest absolute and relative reductions in lean mass typically occur. We have updated the Measurements section to reflect this.

The primary objective of the intervention is to evaluate whether structured exercise can attenuate lean mass loss during this period of negative energy balance, rather than during subsequent weight-stable or weight-regain phases. As such, it was essential that the exercise intervention be delivered concurrently with the low energy diet, rather than introduced after weight loss had occurred. We have clarified in the Discussion section that the short intervention duration limits the generalisability of findings.

From a physiological perspective, measurable changes in lean mass, muscle strength and physical function have been consistently observed within 8–12 weeks of combined dietary and exercise interventions, making this a biologically meaningful period over which to assess lean mass changes. Extending the primary endpoint beyond the active weight-loss phase would introduce additional behavioural and metabolic variability associated with food reintroduction and weight stabilisation, which could obscure the specific effects of exercise on lean mass during energy restriction.

The selected duration reflects a pragmatic balance between physiological relevance, participant burden and available funding. While longer-term follow-up would be helpful for assessing maintenance of lean mass and behavioural changes, this was beyond the scope of this efficacy trial.

3. Explicitly acknowledge the potential bias introduced by the open-label design and greater structured contact in the exercise arm, and clarify any strategies to mitigate or monitor this.

Response:

We agree that the open-label design and the greater structured contact with exercise physiologists in the exercise arm represent potential sources of bias, which are inherent to lifestyle intervention trials and cannot be fully eliminated.

This limitation has been explicitly acknowledged in the Discussion. To mitigate potential bias in outcome assessment, all primary and secondary endpoints are measured using objective, validated methods, with blinded endpoint assessment and laboratory analyses wherever possible.

We have revised the Discussion section with an additional paragraph outlining these limitations in more detail:

This study has several limitations that should be considered. The open-label design may allow changes in participant behaviour that are not fully attributable to the prescribed interventions, and differential contact between groups, introduced through supervised exercise sessions, may introduce bias. These risks are partially mitigated through the use of objective, validated outcome measures and blinded assessment of primary endpoints and laboratory analyses. It is also possible that participants allocated to the low energy diet only group independently alter physical activity behaviours during the intervention period; however, objectively measured accelerometer data are collected to characterise habitual activity and monitor changes over time. In addition, the single-centre setting, relatively short intervention duration, and limited sample size may limit the generalisability of findings, preclude evaluation of longer-term behavioural or physiological maintenance, and lead to type 2 errors in secondary outcomes.

4. Please clarify how ethnicity is determined and whether subgroup analyses or contextual considerations (e.g., nationality differences) will be explored.

Response:

Ethnicity is a complex and multifaceted social construct that encompasses cultural, geographic and ancestral dimensions that cannot be determined with an objective assessment. In this study, ethnicity is assessed through participant self-declaration, consistent with standard data capture practice in UK health research and clinical datasets. Participants are additionally able to identify their South Asian sub-classification (for example: Indian or Bangladeshi origin). We have now added this in the Eligibility criteria section.

Given the mechanistic and physiological focus of the trial, together with the modest sample size, no subgroup analyses beyond sex and predefined weight-loss thresholds are actively planned. Further stratification by nationality or other contextual factors is unlikely to be adequately powered and not expected to materially influence the primary outcome; such analyses are therefore reserved for future larger-scale studies.

5. The analysis plan is broadly appropriate but would benefit from brief clarification regarding handling of missing data (particularly given small N and modified ITT), limitations of multiple imputation, and acknowledgement that secondary outcomes are exploratory and underpowered.

Response: We have added our full Statistical Analysis Plan into the Supplementary section and made reference to this in text. A copy of this document will be uploaded to the ISCRTN Registry as outlined in the Trial Status section. Our pre-specified secondary outcomes are related to the primary outcome and not exploratory but they may however be underpowered. We have noted this in the main text and highlighted this in the Strength and limitations of this study section

References

1. Taylor, R. et al. Aetiology of Type 2 diabetes in people with a ‘normal’ body mass index: testing the personal fat threshold hypothesis. Clinical Science 137, 1333–1346 (2023).
